# Therapeutic Targets for Pediatric Pulmonary Vein Stenosis: Insights from Animal Models

**DOI:** 10.3390/children13050677

**Published:** 2026-05-14

**Authors:** Siqi She, Debao Li, Qi Sun, Lincai Ye

**Affiliations:** 1 Shanghai Institute for Pediatric Congenital Heart Disease, Shanghai Children’s Medical Center, Shanghai Jiao Tong University School of Medicine, Shanghai 200127, China; shesiqi0327@163.com; 2 Department of Pediatric Surgery, Children’s Hospital of Fudan University, National Children’s Medical Center, Shanghai 201102, China; lidebao@sjtu.edu.cn; 3 Department of Thoracic and Cardiovascular Surgery, Shanghai Children’s Medical Center, Shanghai Jiao Tong University School of Medicine, Shanghai 200127, China; empyrealheights@163.com; 4 Institute of Pediatric Translational Medicine, Shanghai Children’s Medical Center, Shanghai Jiao Tong University School of Medicine, Shanghai 200127, China

**Keywords:** pulmonary vein stenosis, animal models, therapeutic targets, sirolimus, losartan, myofibroblasts, translational research

## Abstract

**Highlights:**

**What are the main findings?**

- The evolution from large animal (piglet) models to the neonatal rat bilateral pulmonary vein banding model has enabled reproducible, high-throughput investigation of pediatric pulmonary vein stenosis (PVS) pathophysiology and therapeutic screening.
- Single-cell RNA sequencing and pharmacological studies have identified a pathogenic myofibroblast subpopulation (FAP^+^_Myofib) and revealed a multi-pathway network involving mTOR, TGF-β, YAP/β-catenin, and mechanosensitive signaling (e.g., PIEZO1) as key drivers of PVS progression.

**What are the implications of the main findings?**

- The availability of a cost-effective, genetically tractable neonatal rat model provides a platform for the preclinical evaluation of combination therapies, local drug delivery strategies, and personalized medicine approaches in PVS.
- Targeting newly identified nodes such as FAP, YAP, and mechanosensitive pathways, alongside established targets like mTOR and TGF-β signaling, offers a promising multi-pronged therapeutic strategy to prevent restenosis and disease progression in pediatric PVS.

**Abstract:**

Pulmonary vein stenosis (PVS) is a rare and devastating condition affecting infants and children, characterized by progressive intimal hyperplasia, myofibroblast proliferation, and extracellular matrix deposition, leading to pulmonary hypertension and right heart failure. Despite multimodal interventions including surgery and catheter-based approaches, long-term outcomes remain poor due to high rates of restenosis and disease progression. The development of representative animal models has been instrumental in unraveling the complex pathophysiology of PVS and identifying potential therapeutic targets. This review comprehensively examines the evolution of PVS animal models—from large animals to recently established rodent models—and synthesizes insights gained regarding key pathogenic pathways and their therapeutic implications in guiding associated clinical trials in pediatric patients. We discuss evidence supporting mammalian target of rapamycin (mTOR) inhibition, TGF-β, platelet-derived growth factor (PDGF) and vascular endothelial growth factor (VEGF) targeting, and emerging strategies including fibroblast activation protein (FAP) inhibition and YAP/β-catenin pathway modulation. The recent development of neonatal rat PVS models has accelerated translational research by enabling cost-effective, high-throughput evaluation of candidate therapies. We propose a mechanistic framework integrating these pathways and discuss future directions for precision medicine approaches in PVS.

## 1. Introduction

Pulmonary vein stenosis (PVS) is a rare but highly lethal congenital cardiovascular disorder characterized by progressive narrowing of the pulmonary veins, leading to pulmonary venous hypertension, secondary pulmonary arterial hypertension, and ultimately right heart failure [1,2,3]. The disease predominantly affects infants and young children, with an untreated 2-year mortality rate approaching 60% [4,5]. Despite significant advances in surgical techniques and interventional cardiology over the past two decades, PVS remains one of the most challenging conditions in pediatric cardiology—a sentiment captured by Dr. Prieto LR’s characterization of PVS treatment as the “Holy Grail” of the field [6,7,8,9].

The clinical management of PVS is complicated by two fundamental features of the disease. First, the pathological process is not confined to the main pulmonary veins but diffusely involves distal intraparenchymal vessels, characterized by progressive myofibroblast proliferation and excessive extracellular matrix (ECM) deposition [10,11]. Second, regardless of the intervention strategy—whether surgical resection, sutureless repair, or stent angioplasty—the risk of restenosis remains extraordinarily high, often affecting previously normal veins and leading to inexorable disease progression [12,13,14].

The development of effective targeted therapies for PVS has been hampered by a limited understanding of its underlying mechanisms. Unlike acquired venous stenosis in adults (e.g., post-ablation PVS), pediatric PVS appears to be a distinct entity driven by intrinsic cellular proliferative programs rather than merely a response to mechanical injury [15]. Histopathological studies have established that the neointimal lesions in PVS are composed primarily of myofibroblasts—cells co-expressing α-smooth muscle actin (α-SMA) and vimentin—with variable contributions from endothelial cells, smooth muscle cells, and inflammatory cells [16,17]. However, the origins of these pathogenic myofibroblasts, the signals that drive their proliferation and matrix-producing phenotype, and the reasons for the relentless progression of the disease have remained elusive [18].

In this context, animal models have emerged as indispensable tools for dissecting PVS pathophysiology and evaluating potential therapeutic interventions. This review provides a comprehensive synthesis of insights gained from PVS animal models regarding disease mechanisms and therapeutic targets. We trace the evolution of these models from large animals to the recently developed rodent systems, examine the evidence for various targeted therapies, and propose an integrated framework for future therapeutic development.

## 2. The Evolution of PVS Animal Models

### 2.1. Large Animal Models: Pioneering Contributions and Limitations

The first reproducible animal model of PVS was developed by LaBourne and colleagues in 1990 using neonatal pigs [19]. This model employed surgical banding of the pulmonary veins—a technique inspired by clinical observations that congenital PVS often involves focal narrowing at the venoatrial junction [20]. By placing constrictive bands around individual pulmonary veins in 3–4-week-old piglets, the investigators successfully recapitulated key features of human PVS, including progressive intimal hyperplasia, medial hypertrophy, and ECM remodeling. Hemodynamic assessments confirmed the development of pulmonary hypertension, and ultrastructural studies revealed alterations in elastin and collagen content within the stenotic vessels [19].

The neonatal pig model offered several advantages: the cardiovascular system of young pigs closely resembles that of human infants; the size of the animals permits detailed hemodynamic and imaging studies; and the rapid growth of piglets (reaching adult size within months) allows for the observation of progressive pathology over a compressed timeframe [21,22]. Subsequent refinements of this model by multiple groups have provided critical insights into PVS pathogenesis.

Kato and colleagues [18] used the piglet PVS model to investigate the phenomenon of “upstream” vasculopathy—the extension of pathological changes into pulmonary veins distant from the primary stenotic site. They demonstrated that endothelial–mesenchymal transition (EndMT) plays a central role in this process, with endothelial cells acquiring mesenchymal characteristics and contributing to the expanding pool of neointimal myofibroblasts. This observation provided a mechanistic explanation for the diffuse nature of PVS and the tendency for disease progression even after successful relief of focal obstructions.

The piglet model also enabled the first preclinical evaluation of targeted therapy for PVS. Zhu et al. [23] demonstrated that treatment with losartan, an angiotensin II type 1 receptor (AT1R) blocker, attenuated neointimal hyperplasia and improved hemodynamic parameters in banded animals. Mechanistically, losartan treatment was associated with reduced expression of transforming growth factor-β1 (TGF-β1) and markers of EndMT, supporting a role for TGF-β/EndMT-associated signaling in pulmonary venous remodeling [23]. This work provided the rationale for evaluating losartan as a potential modulator of TGF-β/EndMT-associated remodeling in pediatric PVS.

Masaki and colleagues [24] further refined the piglet model by performing pulmonary vein transection and re-anastomosis, mimicking the surgical repair of total anomalous pulmonary venous connection. This model revealed progressive vascular remodeling extending into the intraparenchymal veins and confirmed the involvement of TGF-β signaling and EndMT in the pathogenesis of post-repair PVS.

Despite these invaluable contributions, large animal models have significant limitations that constrain their utility for therapeutic discovery. The cost of procuring and housing pigs is prohibitive for most academic laboratories (approximately ¥5000–10,000 per animal), and the 8–12 week time course required for disease development limits experimental throughput [19,23,24]. The complexity of the surgical procedures demands specialized expertise and equipment, and the genetic heterogeneity of outbred pig populations introduces variability that complicates mechanistic studies. Most importantly, the scale and expense of pig studies preclude their use for high-throughput drug screening or systematic evaluation of multiple therapeutic candidates.

### 2.2. Rodent Models: Recent Technical Advances

The development of rodent models for PVS has represented an important methodological advance in the field. Mice and rats offer practical advantages for translational research: low cost (approximately 1/100th that of pigs), rapid reproduction, more standardized experimental backgrounds, and greater experimental flexibility [25,26,27,28]. However, the technical challenges of performing pulmonary vein surgery in neonatal rodents—whose pulmonary veins measure less than 0.3 mm in diameter—remain substantial and require a high level of microsurgical precision.

#### 2.2.1. Neonatal Rat PVS Models

The neonatal rat PVS models described in this subsection were developed by Li and colleagues, including members of the present author group [29,30]. Following extensive refinement of microsurgical techniques for neonatal cardiovascular surgery, this group first reported a unilateral PVS model in 2023 [30]. In that model, left pulmonary vein banding was performed in 1–3-day-old rat pups under microscopic guidance, resulting in progressive venous stenosis that reproduced several hallmark pathological features of human PVS, including neointimal hyperplasia, myofibroblast accumulation, and ECM deposition [10].

**Figure 1:**
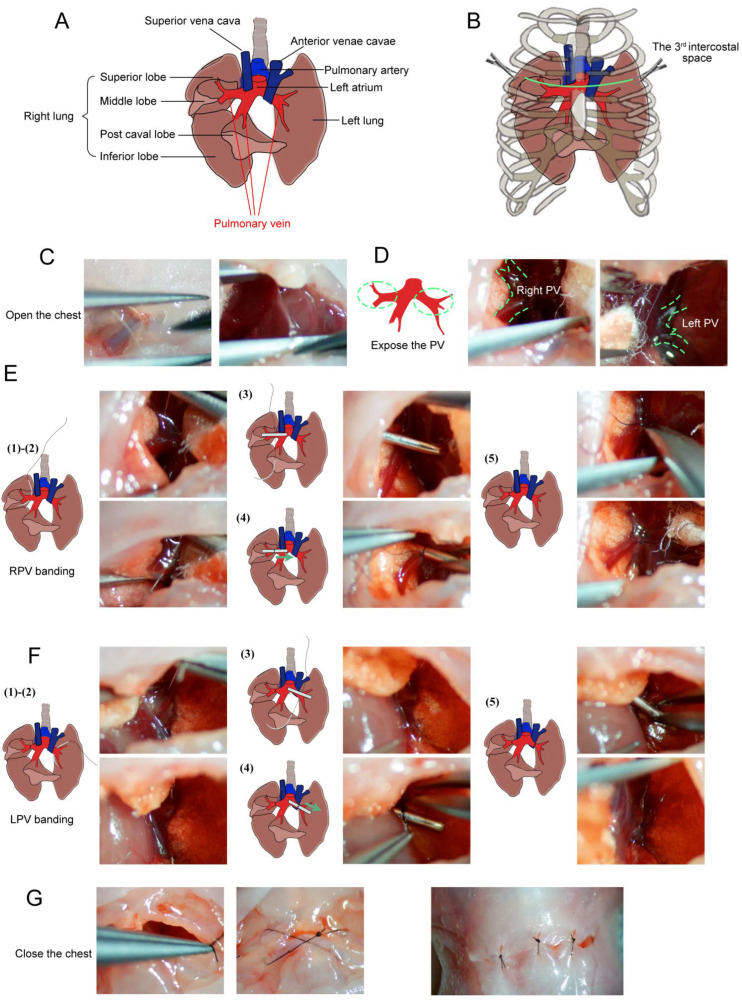
Schematic and microscopic views of bilateral pulmonary vein (PV) banding surgical procedures. (**A**) Schematic diagram of the local anatomical structures around the pulmonary veins (PVs). (**B**) Schematic diagram of exposed PVs between the left 3rd and 4th ribs (green line) with minimal incision. (**C**) The sternum was cut, and the chest opened. (**D**) Exposed left and right PVs. (**E**) Right PV (RPV) banding: (1) A blunt needle is placed beside the RPV; (2) Needle is passed below the RPV; (3) Placement of the 28G padding needle; (4) Padding needle is ligated with the RPV; (5) Removal of the padding needle and cutting of the end of the sutures. (**F**) Left PV (LPV) banding: (1) A blunt needle beside the LPV; (2) Needle is passed below the LPV; (3) Placement of the 28G padding needle; (4) Padding is needle ligated with the LPV; (5) Removal of the padding needle and cutting of the end of the sutures. (**G**) Layer-by-layer skin closure. Schematic views are shown on the left; microscopic images are shown on the right (adapted from [29] under the CC BY license).

However, the unilateral model had limitations: approximately 30% of animals developed complete venous occlusion with collateral vessel formation, and the pathological changes were confined to the banded side. In 2025, the same group introduced a modified bilateral PVS model that addressed these issues [7,29]. By banding both the right and left pulmonary veins with a standardized constriction (using a 30G needle as a spacer to ensure consistent lumen diameter) (Figure 1), they achieved stable, reproducible bilateral stenosis with less than 10% collateralization. The model faithfully recapitulated the clinical progression of PVS, with echocardiographic evidence of increased flow velocity at banding sites, reduced pulmonary artery acceleration time (indicating pulmonary hypertension), elevated right ventricular pressure, and right ventricular hypertrophy by postnatal day 21–30 [29].

The utility of this model for therapeutic evaluation was demonstrated through studies of sirolimus (rapamycin), an mTOR inhibitor that had shown promise in single-center clinical studies [31,32,33]. Treatment of banded pups with sirolimus (1.5 mg/kg every 3 days from postnatal day 7) significantly reduced neointimal thickness and the number of α-SMA-positive myofibroblasts, while preserving lumen diameter [29]. Importantly, these studies also revealed the potential limitations of sirolimus therapy: treated animals showed reduced body weight gain, suggesting that the drug’s anti-proliferative effects might adversely affect somatic growth in infants [29]. This finding has important implications for clinical practice, highlighting the need for careful risk–benefit assessment and potentially for the development of more targeted therapies with fewer off-target effects.

**Figure 2:**
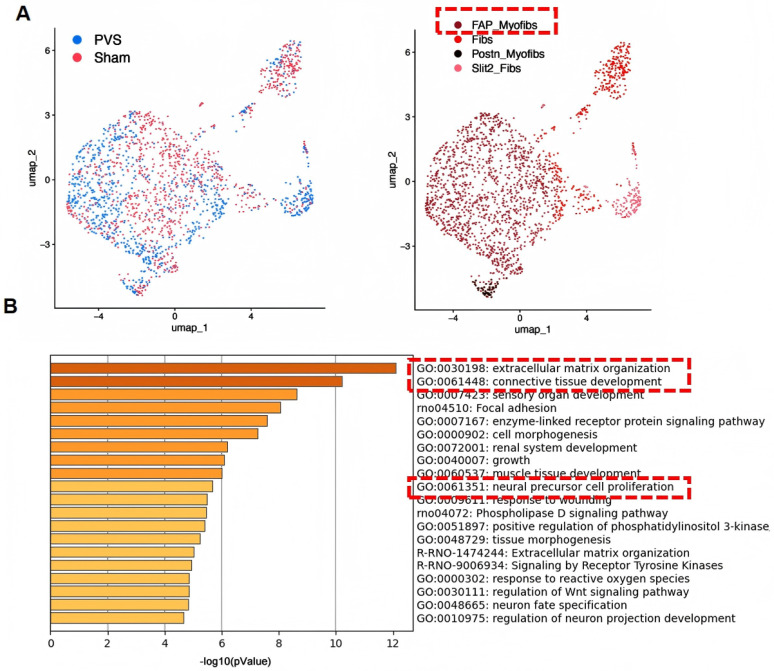
Single-cell RNA sequencing reveals the heterogeneity of myofibroblasts in PVS. (**A**) FAP_Myofib is the predominant subset in PVS. (**B**) Enrichment analysis of genes highly expressed in FAP_Myofib reveals their molecular characteristics associated with extracellular matrix secretion and proliferation.The red box in panel A highlights the FAP_Myofib subpopulation, while the red boxes in panel B highlight representative enriched pathways related to extracellular matrix remodeling and cellular proliferation (adapted from Supplementary Figure S3 of Li et al. [29] under the CC BY license).

The neonatal rat model has also enabled transcriptomic analyses that were previously impossible in large animal systems. Bulk RNA sequencing of stenotic pulmonary veins revealed differential expression of genes involved in ECM remodeling, cell cycle regulation, and inflammatory pathways, providing a rich resource for hypothesis generation [29]. In the same published study, single-cell RNA sequencing further refined our understanding of PVS cellular heterogeneity, identifying distinct myofibroblast subpopulations with unique functional characteristics (Figure 2).

#### 2.2.2. PH-LHD Rat Models

Parallel efforts have focused on modeling pulmonary hypertension due to left heart disease (PH-LHD), a related condition that shares features with PVS [34,35]. Earlier swine pulmonary vein banding models demonstrated that chronic pulmonary venous obstruction can induce postcapillary pulmonary hypertension, right ventricular remodeling, and secondary pulmonary vascular remodeling [36]. Subsequent work further linked this progression to endothelin signaling and showed that isolated postcapillary pulmonary hypertension may evolve into combined precapillary and postcapillary pulmonary hypertension [37].

More recently, Shentu and colleagues [38] developed a rat model of pulmonary venous congestion by performing two-stage surgical banding of all pulmonary veins. At 8 weeks post-surgery, animals developed severe pulmonary hypertension (mean pulmonary artery systolic pressure of 80 mmHg), right ventricular hypertrophy (Fulton’s index 0.7), and progressive vascular remodeling. Multi-omics analysis revealed 11 commonly enriched pathways in the lung, including ECM-receptor interaction, focal adhesion, and PPAR signaling [38], providing potential therapeutic targets for PH-LHD that may be relevant to PVS.

### 2.3. Genetic Models and Future Directions

The development of genetic mouse models for PVS has been challenging due to the embryonic lethality of mutations affecting vascular development [39,40]. However, conditional approaches may eventually enable tissue-specific manipulation of candidate genes in the postnatal period. The availability of well-characterized neonatal rat surgical models now provides a platform for evaluating the functional significance of genes identified through human genetics or transcriptomic studies, using approaches such as in vivo siRNA knockdown or CRISPR-mediated gene editing.

## 3. Therapeutic Targets Identified Through Animal Models

### 3.1. mTOR Signaling and Sirolimus

The mammalian target of rapamycin (mTOR) is a serine/threonine kinase that integrates signals from growth factors, nutrients, and cellular energy status to regulate cell growth, proliferation, and metabolism [41,42,43]. The rationale for targeting mTOR in PVS stems from the observation that the neointimal lesions resemble benign myofibroblastic tumors, and from the success of mTOR inhibitors in treating other proliferative vascular conditions such as coronary artery restenosis [10,44].

Preclinical evidence for mTOR inhibition in PVS has been obtained from both large-animal surgical models and the neonatal rat model. Masaki and colleagues [24] demonstrated activation of mTOR signaling and therapeutic benefit from rapamycin, eluting films in a large-animal surgical model. Li et al. [29] demonstrated that sirolimus treatment significantly reduced neointimal hyperplasia and preserved lumen diameter in banded pulmonary veins. Histological analysis revealed decreased numbers of α-SMA-positive myofibroblasts and reduced collagen deposition, consistent with inhibition of both cellular proliferation and matrix production [10,18]. Importantly, the therapeutic effect was observed even when treatment was initiated after stenosis had already developed, suggesting potential utility for established disease [29].

Transcriptomic analysis of sirolimus-treated animals provided mechanistic insights. Differentially expressed genes in response to treatment included those involved in cell cycle regulation (e.g., cyclins, CDKs), ECM remodeling (matrix metalloproteinases, collagens), and inflammatory pathways [29]. Pathway analysis suggested that sirolimus exerts its effects not only through direct inhibition of myofibroblast proliferation but also by modulating the inflammatory microenvironment and ECM turnover [29,45].

Clinical studies have provided support for these preclinical findings. Patel and colleagues [31] reported that systemic sirolimus therapy in infants with severe, multi-vessel PVS was associated with improved 2-year survival compared to historical controls. However, interpretation of these results is complicated by the concurrent use of frequent stent placements and the absence of a randomized control group [46]. A subsequent multicenter study confirmed that sirolimus treatment was associated with reduced intervention frequency, but also highlighted significant variability in response among patients and even among individual veins within the same patient [32].

The observation that sirolimus inhibits somatic growth in neonatal rats raises important safety considerations for clinical use [29,43,47]. Growth suppression is a well-recognized effect of mTOR inhibitors in pediatric populations, reflecting the critical role of mTOR signaling in normal development [48]. This finding underscores the need for strategies to maximize therapeutic efficacy while minimizing off-target effects, such as local drug delivery (e.g., drug-eluting stents) or combination therapy with lower doses of multiple agents.

### 3.2. TGF-β Signaling and Losartan

Losartan is an angiotensin II type 1 receptor blocker with anti-remodeling effects that include attenuation of TGF-β-associated signaling in experimental cardiovascular models [49,50]. In PVS, piglet studies linked upstream pulmonary venous remodeling to TGF-β/Smad signaling and EndMT-related phenotypic changes, providing the rationale for evaluating losartan as a modulator of pulmonary venous remodeling [18,23].

Zhu and colleagues [23] first reported that losartan treatment attenuated neointimal hyperplasia in banded piglets and reduced expression of TGF-β1 and markers of EndMT. These findings were extended by subsequent studies demonstrating that losartan also improved endothelial function in upstream pulmonary veins, partially restoring endothelium-dependent relaxation [51]. Mechanistic studies suggested that these effects were mediated in part by reduction in NADPH oxidase (NOX)-derived reactive oxygen species, which contribute to endothelial dysfunction and eNOS uncoupling [51,52]. Collectively, these findings suggest that losartan may attenuate TGF-β-associated EndMT and endothelial dysfunction in experimental PVS.

However, clinical translation of losartan for pediatric PVS remains incomplete. A phase I/II pilot study of losartan in pediatric PVS (NCT02769130) was initiated to evaluate safety and feasibility, but its progress was interrupted in the context of broader losartan formulation recalls related to nitrosamine impurity concerns [53,54,55]. This interruption should therefore not be interpreted as evidence of therapeutic failure, but rather as a reminder that animal-model findings require careful validation and controlled clinical translation.

Subsequent work has suggested that losartan may exert its effects through pathways beyond TGF-β inhibition [56]. Zeng and colleagues [56] demonstrated in a modified piglet model that losartan treatment attenuated upstream vasculopathy and was associated with modulation of the Hippo signaling pathway, specifically increased phosphorylation (inactivation) of YAP. This finding suggests that losartan may target a convergence point for multiple pro-fibrotic signals and has stimulated interest in directly targeting YAP/TAZ as a therapeutic strategy [57,58].

### 3.3. PDGF and VEGF Pathways

Platelet-derived growth factor (PDGF) and vascular endothelial growth factor (VEGF) are receptor tyrosine kinase pathways that regulate cell proliferation, migration, and survival. The rationale for targeting these pathways in PVS derives from immunohistochemical studies demonstrating expression of PDGF receptors and VEGF receptors in PVS lesions [16,59].

Riedlinger and colleagues [16] first reported expression of receptor tyrosine kinases—including PDGFR-β and VEGFR-2—by neointimal cells in human PVS specimens. Based on this observation, Callahan and colleagues [59] conducted a phase II trial of adjunctive biologic inhibition agents targeting PDGF and VEGF in children with aggressive multi-vessel PVS. Patients received imatinib (a PDGFR inhibitor) or bevacizumab (a VEGF inhibitor) in addition to standard therapy. While some patients showed stabilization of disease, the overall results were mixed, and the study was limited by small sample size and lack of a control group.

Preclinical evaluation of imatinib in the neonatal rat PVS model has not yet been reported, but such studies could provide valuable information about the potential efficacy of this approach and help identify biomarkers predictive of response. The availability of a cost-effective rodent model now makes it feasible to systematically evaluate multiple tyrosine kinase inhibitors and to test combination strategies.

### 3.4. Emerging Targets: Mechanosensitive Signaling, YAP/β-Catenin, and FAP

Endothelial and mural cells are subjected to altered mechanical forces during pulmonary venous obstruction, including changes in wall shear stress, pressure, and matrix stiffness [60]. These mechanical cues may be transduced into intracellular signaling programs that regulate proliferation, mesenchymal transition, and matrix remodeling [57,58]. Mechanotransduction pathways have therefore been increasingly implicated in pulmonary venous remodeling in experimental models and human PVS.

The Hippo–YAP/β-catenin pathway has emerged as a candidate mechanosensitive signaling axis in pulmonary venous remodeling. YAP and its paralog TAZ function as transcriptional coactivators that regulate proliferation, differentiation, and tissue remodeling in response to mechanical and soluble signals [57,58]. In the piglet PVS model, YAP activation was observed in stenotic pulmonary veins, and losartan treatment was shown to increase YAP phosphorylation, consistent with YAP inactivation [56]. In vitro, angiotensin II stimulation was found to induce YAP nuclear translocation and transcriptional activity in endothelial cells, effects that were blocked by losartan [56,61].

The relevance of mechanosensitive signaling in aggressive PVS is further supported by transcriptomic data from patient tissue. RNA sequencing of pulmonary vein specimens from pediatric PVS patients revealed increased PIEZO1 expression as a potential feature of aggressive disease [62]. Because PIEZO1 encodes a mechanically activated ion channel, these observations warrant continued investigation of mechanosensitive signaling in complementary preclinical models, although the functional role of PIEZO1 in PVS remains to be established.

Recent advances in single-cell technologies have enabled unprecedented resolution of PVS cellular heterogeneity and have revealed novel therapeutic targets. Single-cell RNA sequencing of stenotic pulmonary veins from the neonatal rat model identified FAP (fibroblast activation protein) as a highly specific marker of a pathogenic myofibroblast subpopulation (FAP_Myofibs) (Figure 2, pending patent CN2025101268665) that exhibits dual functional characteristics: high proliferative activity and strong engagement of ECM remodeling pathways.

FAP is a type II transmembrane serine protease with both dipeptidyl peptidase and endopeptidase activities that cleave Gly-Pro motifs in ECM proteins, releasing bioactive peptides that can activate multiple downstream pathways including TGF-β [63,64,65]. While FAP represents a promising target based on our scRNA-seq and preliminary pharmacological data, its role in PVS requires further validation in independent studies and peer-reviewed publications.

### 3.5. Iron Metabolism and Ferroptosis: Lessons from PVOD

Although distinct from PVS, pulmonary veno-occlusive disease (PVOD) shares features of pulmonary venous remodeling and has provided insights that may be relevant to PVS [66]. Recent work by Zhang and colleagues [66] demonstrated that ferroptosis—an iron-dependent form of regulated cell death—plays a critical role in PVOD pathogenesis. Using a mouse model of GCN2 deficiency (which recapitulates familial PVOD), they showed that macrophage ferroptosis releases iron that drives pulmonary venous endothelial arterialization through ETS1-mediated upregulation of arterial-specific genes [66,67]. Treatment with the ferroptosis inhibitor Ferrostatin-1 effectively prevented and even reversed disease progression in both genetic and toxin-induced models [67].

While PVS and PVOD have different etiologies and histological features, both involve pathological venous remodeling and myofibroblast accumulation. The possibility that iron metabolism or ferroptosis contributes to PVS pathogenesis merits investigation, and the availability of the neonatal rat PVS model provides a platform for testing ferroptosis inhibitors in this context.

## 4. Mechanistic Insights from Animal Models

**Figure 3:**
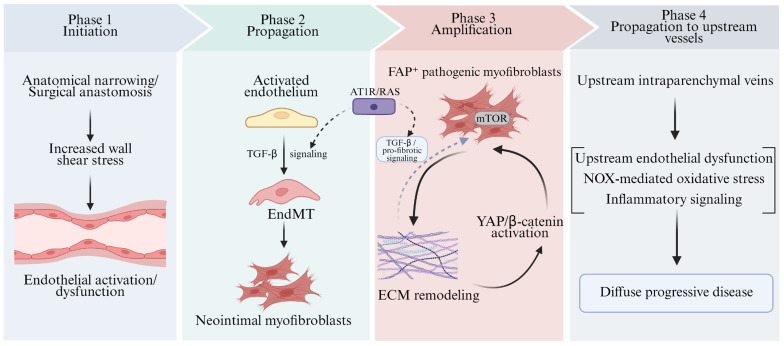
Mechanistic insights into pediatric pulmonary vein stenosis derived from animal models. This schematic summarizes model-derived mechanisms implicated in pediatric PVS progression. Solid arrows indicate relationships supported by available preclinical evidence, whereas dashed arrows denote putative modulatory or integrative links. TGF-βbeta: Transforming Growth Factor-beta; EndMT: Endothelial-to-Mesenchymal Transition; mTOR: mechanistic Target of Rapamycin; ECM: Extracellular Matrix; YAP: Yes-associated Protein; FAP: Fibroblast Activation Protein; NOX: NADPH Oxidase.

Studies in large-animal and rodent models have identified several recurring mechanisms that may contribute to pediatric PVS pathobiology. The neonatal piglet model first demonstrated that experimentally induced pulmonary venous obstruction could reproduce key features of progressive vascular remodeling, including intimal hyperplasia and alterations in extracellular matrix components such as elastin and collagen [19]. Subsequent piglet studies provided evidence that pathological remodeling may extend into upstream pulmonary veins and implicated endothelial-to-mesenchymal transition (EndMT) and TGF-β signaling in lesion propagation [18,23,24]. These findings are consistent with clinical observations suggesting that elevated wall shear stress may contribute to PVS progression [60,68] and with broader cardiovascular evidence implicating TGF-β-regulated EndMT in vascular remodeling [69]. Neonatal rat PVS models provide a complementary platform for examining neointimal expansion, myofibroblast accumulation, extracellular matrix deposition, and pharmacologic responses to candidate therapies, including sirolimus [29,30]. Additional preclinical work in the piglet model has suggested that mechanotransduction-related pathways, including the Hippo–YAP pathway, may also contribute to pulmonary venous remodeling [56]. Collectively, findings from large-animal and rodent models support a multifaceted framework of pediatric PVS pathogenesis, in which endothelial injury, mesenchymal transition, myofibroblast proliferation, extracellular matrix remodeling, and mechanotransduction-related signaling may act in concert to promote disease progression (Figure 3). Table 1 summarizes representative therapeutic targets and candidate strategies informed by these animal model findings.

## 5. Choosing the Right Model for the Right Question

The availability of multiple PVS animal models raises the question of how to select the appropriate model for specific research questions. Table 2 summarizes the key features, advantages, and limitations of currently available models.

The piglet model remains the gold standard for detailed hemodynamic and imaging studies, for evaluation of surgical interventions, and for final preclinical validation of promising therapies. Its anatomical similarity to humans and the ability to perform comprehensive cardiac catheterization and sophisticated imaging make it indispensable for certain questions.

The neonatal rat model is well suited for mechanistic studies requiring genetic manipulation, for high-throughput screening of therapeutic candidates, and for studies requiring large sample sizes (e.g., dose–response studies, combination therapy optimization). The recent development of bilateral PVS models with improved reproducibility has further enhanced their utility.

Emerging models, such as the PH-LHD rat model [34] and potentially genetic mouse models, will address specific aspects of PVS pathophysiology and enable studies not possible in surgical models (e.g., investigation of developmental origins).

## 6. Translating Insights from Animals to Patients

The ultimate goal of animal model research is to improve outcomes for patients with PVS [70,71]. The path from preclinical discovery to clinical application requires careful validation at multiple levels and awareness of the limitations of animal models [71].

### 6.1. Successes and Limitations of Translation

The translational trajectory of sirolimus illustrates both the promise and challenges of this process. Preclinical studies in the neonatal rat model have provided clear evidence of efficacy and also identified potential safety concerns (growth suppression) [29]. Clinical studies have suggested a benefit but have been limited by small sample sizes, a lack of randomization, and confounding by concomitant interventions [31,32]. The heterogeneity of PVS—with variation in disease aggressiveness, response to treatment, and natural history among patients and even among individual veins—complicates clinical trial design and interpretation [72].

The experience with losartan highlights the importance of rigorous preclinical validation and careful clinical translation. Despite promising results in piglet models and a strong mechanistic rationale, the phase I/II pilot trial of losartan in pediatric PVS (NCT02769130) was temporarily suspended in the context of formulation-related recall and protocol modification [23,51]. This interruption illustrates that translation from animal models to patients requires not only mechanistic efficacy, but also careful control of drug formulation, trial design, and metabolic differences. Potential challenges include species differences in drug metabolism, differences in disease pathogenesis between surgical models and human disease.

### 6.2. Towards Precision Medicine in PVS

The identification of distinct myofibroblast subpopulations through single-cell profiling raises the possibility of precision medicine approaches in PVS. Patients with high abundance of FAP_Myofibs might be particularly responsive to FAP-targeted therapies; those with evidence of YAP pathway activation might benefit from YAP inhibitors; and those with prominent inflammatory signatures might respond to anti-inflammatory agents [73,74].

The development of biomarkers to stratify patients and predict treatment response is a priority [75]. Candidate biomarkers include circulating levels of FAP or its cleavage products, imaging markers of disease activity (e.g., PET tracers targeting FAP), and genetic variants influencing drug metabolism or disease susceptibility. The availability of well-annotated biobanks from patients undergoing surgical resection or transplantation will be essential for biomarker validation.

## 7. Conclusions

Animal models have substantially advanced our understanding of pediatric PVS by faithfully recapitulating key features of pulmonary venous obstruction, upstream vascular remodeling, and fibroproliferative lesion formation. Piglet models remain valuable for investigating hemodynamic injury and upstream pulmonary venous remodeling, while neonatal rat models provide a scalable platform for assessing neointimal expansion, extracellular matrix deposition, and pharmacological responses to candidate therapies. Collectively, these complementary models have enabled the identification of candidate mechanisms and therapeutic targets warranting further investigation. However, as no single model fully captures the biological heterogeneity of human pediatric PVS, preclinical findings should be interpreted with caution. Ongoing validation across complementary animal models and human tissue specimens remains essential before insights derived from animal studies can be translated into clinical practice.

## Figures and Tables

**Table 1:** Animal model-derived mechanisms and candidate intervention points in pediatric PVS.

Phase	Main Therapeutic Target (s)	Representative Therapeutic Strategy
1. Initiation	Endothelial activation/dysfunction	Limiting endothelial injury and downstream remodeling
2. Propagation	TGF-β/EndMT axis	Preventing EndMT (e.g., TGF-β inhibitors, losartan)
3. Amplification	Myofibroblast proliferation and ECM remodeling	Inhibiting myofibroblast proliferation (e.g., sirolimus, imatinib) and blocking ECM remodeling/feedback amplification (e.g., FAP inhibitors)
4. Propagation to upstream vessels	Progressive vascular remodeling	Targeting mechanotransduction pathways (e.g., YAP inhibitors)

**Table 2:** Comparison of PVS Animal Models.

Feature	Piglet Model	Neonatal Rat Model
Cost per animal experiment	¥5000–10,000	¥50–100
Time to disease	8–12 weeks	3–4 weeks
Surgical complexity	High	Very high (microsurgery)
Genetic tools	Limited	Available
Throughput	Low	High
Hemodynamic assessment	Comprehensive	Limited by size
Human relevance	High (anatomy)	High (pathology)

## Data Availability

The original contributions presented in this study are included in the article/Supplementary Material. Further inquiries can be directed to the corresponding author.

## Supplementary Materials

The following supporting information can be downloaded at: https://www.mdpi.com/article/10.3390/children13050677/s1.
